# Computational Models on Pathological Redox Signalling Driven by Pregnancy: A Review

**DOI:** 10.3390/antiox11030585

**Published:** 2022-03-18

**Authors:** Samprikta Manna, Camino S. M. Ruano, Jana-Charlotte Hegenbarth, Daniel Vaiman, Shailendra Gupta, Fergus P. McCarthy, Céline Méhats, Cathal McCarthy, Clara Apicella, Julia Scheel

**Affiliations:** 1 Department of Obstetrics and Gynaecology, Cork University Maternity Hospital, University College Cork, T12 YE02 Cork, Ireland; fergus.mccarthy@ucc.ie; 2 Institut Cochin, Inserm U1016, UMR8104 CNRS, Université de Paris, 75014 Paris, France; camino.ruano@inserm.fr (C.S.M.R.); daniel.vaiman@inserm.fr (D.V.); celine.mehats@inserm.fr (C.M.); clara.apicella@inserm.fr (C.A.); 3 Department of Molecular Genetics, Faculty of Science and Engineering, Faculty of Health, Medicine and Life Sciences, Maastricht University, 6211 KH Maastricht, The Netherlands; j.hegenbarth@maastrichtuniversity.nl; 4 Department of Cardiology, CARIM School for Cardiovascular Diseases, Faculty of Health, Medicine and Life Sciences, Maastricht University, 6229 ER Maastricht, The Netherlands; 5 Department of Systems Biology and Bioinformatics, Rostock University, 18051 Rostock, Germany; shailendra.gupta@uni-rostock.de (S.G.); julia.scheel@uni-rostock.de (J.S.); 6 Department of Pharmacology and Therapeutics, Western Gateway Building, University College Cork, T12 K8AF Cork, Ireland; cmccarthy@ucc.ie

**Keywords:** oxidative stress, preeclampsia, systems biology, antioxidant therapy, integrative modelling, cardio-obstetrics

## Abstract

Oxidative stress is associated with a myriad of diseases including pregnancy pathologies with long-term cardiovascular repercussions for both the mother and baby. Aberrant redox signalling coupled with deficient antioxidant defence leads to chronic molecular impairment. Abnormal placentation has been considered the primary source for reactive species; however, placental dysfunction has been deemed secondary to maternal cardiovascular maladaptation in pregnancy. While various therapeutic interventions, aimed at combating deregulated oxidative stress during pregnancy have shown promise in experimental models, they often result as inconclusive or detrimental in clinical trials, warranting the need for further research to identify candidates. The strengths and limitations of current experimental methods in redox research are discussed. Assessment of redox status and oxidative stress in experimental models and in clinical practice remains challenging; the state-of-the-art of computational models in this field is presented in this review, comparing static and dynamic models which provide functional information such as protein-protein interactions, as well as the impact of changes in molecular species on the redox-status of the system, respectively. Enhanced knowledge of redox biology in during pregnancy through computational modelling such as generation of Systems Biology Markup Language model which integrates existing models to a larger network in the context of placenta physiology.

## 1. Introduction

Reactive oxygen and nitrogen species (RONS) are recognised as important signalling molecules within cells. Redox signalling occurs when a biological system alters in response to a change in the level of a particular reactive species (RS) or the shift in redox state of a redox-sensitive group. RONS are physiological signalling molecules at low levels but cause oxidative stress (OS) at high levels, resulting in damage to cell organelles, especially mitochondria. The role of OS has been extensively proven in pathological outcomes, including but not limited to aging, diabetes, cancer, and cardiovascular diseases (CVD) [1,2,3,4].

OS is considered a hallmark of pregnancy pathologies such as preeclampsia (PE), defined by de novo hypertension and proteinuria after 20 weeks of gestation, and intrauterine growth restriction (IUGR), a condition where foetal weight at birth falls below the 10th percentile for comparable gestational age [5,6]. Evidence suggests that defective placental implantation causes reduced remodelling of maternal spiral arteries, maintaining high resistance and a contractile phenotype, resulting in reduced blood flow to the placenta and the foetus. The placenta therefore remains abnormally susceptible to fluctuations in blood flow and pressure, leading to ischemic reperfusion injury, with excessive production of RONS and antioxidant imbalance. Toxic by-products are released into the maternal circulation and are detectable biomarkers of OS, which have an impact on the maternal vasculature, as seen in PE through endothelial dysfunction (ED) [7,8]. Predisposition to PE has been linked to the same risk factors for CVD, suggesting an inter-play between the placenta and the cardiovascular system. This is supported by evidence that cardiovascular injury can occur before the development of PE. The inability of the cardiovascular system to sustain the challenge of pregnancy may therefore be the cause of reduced blood flow to the placenta, resulting in pregnancy pathologies [9].

In recent years, experimental data collected from redox studies in a wide variety of model organisms such as bacteria, yeast, plants, and mice have been used to develop computational models and to make inferences on redox signalling in the human system. These models allow us to investigate how changes in stimuli such as growth factors, oxygen tension, RONS and antioxidant levels can impact the fine balance between redox homeostasis and damage, culminating in pathological outcomes [10].

The scope of this review is to summarise the processes involved in redox-signalling and oxidative damage, in the context of pregnancy and cardiovascular regulation, while presenting state-of-the-art redox-specific computational modelling, which could prove fundamental in advancing our understanding of OS in pregnancy and cardio-obstetrics.

### 1.1. Redox Signalling

Redox signalling cascades are based on processes of electron-transfer and involve RS and antioxidant cellular systems, as well as redox-sensitive effector proteins. Downstream of stimuli such as OS, metabolic changes, or inflammation, the controlled and spatially confined production of RS is an important cellular mechanism to generate key second messengers (i.e., H_2_O_2_, NO) that reduce the risk of cellular damage [11,12]. These pathways regulate cell proliferation, autophagy, inflammation, apoptosis, cardiovascular tone regulation and hormone production [13,14,15,16,17].

Effector proteins including kinases, phosphatases, transcription factors, chaperones, and enzymes, partake in reversible redox reactions in the presence of key cysteine residues and respective sulfhydryl groups (-SH), as well as transition metal groups (i.e., selenium, iron, manganese) [18,19,20]. Oxidation or reduction of these functional groups is considered as a post-translational modification (PTM), usually resulting in a conformational change of the effector protein, influencing dimerization and altering its biological activity, specificity and/or stability.

### 1.2. Reactive Species

Mitochondria are the primary source of intracellular reactive oxygen species (ROS). During ATP production by the mitochondria via the electron transport chain (ECT), electron leakage can occur which react with oxygen, generating free radicals’ superoxide •O_2_^−^, hydroxyl HO•, peroxyl radicals ROO•, and non-radical hydrogen peroxide H_2_O_2_ [21,22]. Membrane-embedded NADPH oxidase enzymes (Nox) are the second main source of ROS, generating superoxide from NADPH and oxygen [23]. Dual oxidases (DUOX), DUOX1 and DUOX2, are the sole members of the Nox family to contain an additional, extracellular, peroxidase-like domain that catalyses the extracellular synthesis of H_2_O_2_ [24]. Apart from the mitochondria, ROS production also occurs in peroxisomes, lysosomes, Golgi, and endoplasmic reticulum membranes, by xanthine oxidases during purine degradation, cytochrome P450 is involved in biosynthesis of fatty acids and clearance of xenobiotics [25,26,27,28].

The main reactive nitrogen species (RNS), nitric oxide (NO), is an important signalling molecule involved in PTM, endothelium-mediated vasodilation, angiogenesis, neural development, and immune responses [29,30]. In the presence of ROS, more destructive RNS such as nitrogen dioxide (•NO_2_), dinitrogen trioxide (N_2_O_3_), dinitrogen tetroxide (N_2_O_4_), and peroxynitrite (ONOO^−^) are generated. In the presence of CO_2,_ ONOO^−^ generates •NO_2_ and •HCO_3_, which are highly reactive carbonate radicals and have a strong tendency to interact with proteins and lipids, resulting in damaging nitrosylation and carbonylation [31]. The oxidative modification of cysteine residues by NO to form S-nitrosothiols is called S-nitrosylation, which triggers a change in protein structure, alters protein–protein interactions and enables further PTMs such as phosphorylation, acetylation, ubiquitination, and disulphide bond formation. S-nitrosylation is a tightly regulated, key mechanism through which NO signalling is propagated within the cell, tissue, and microenvironment [31].

NOS are cytoplasmic enzymes that catalyse NO generation from l-arginine, NADPH, and oxygen, when binding to specific cofactors calcium-calmodulin, NADPH, Flavin adenine dinucleotide (FAD), flavin mononucleotide (FMN), heme and tetrahydrobiopterin (BH4). Endothelial (eNOS), neuronal (nNOS) and inducible NOS (iNOS) have been identified as three isoforms of NOS expressed by immune cells [32,33]. When NOS becomes uncoupled from its substrate or cofactors, it generates superoxide [34]. Reactive Sulfur Species (RSS) go beyond the scope of the current review [35].

### 1.3. Antioxidant Cellular Systems

Antioxidant molecules maintain the cell in a reducing environment and are responsible for detoxification of RONS, by partaking in redox reactions, undergoing reversible oxidation, usually at the expense of NADPH [36]. Antioxidant enzymes are found abundantly in the molecular environment. Superoxide dismutase (SOD) catalyses the conversion of superoxide to H_2_O_2_ through a metal group in the active site. Copper and zinc-SOD are found both in the cytosol and extracellularly, whereas manganese-SOD is mitochondrial [37,38,39]. Catalase (CAT) converts H_2_O_2_ into water and oxygen by reduction of the iron atom, at the centre of the heme group in the active site [40,41]. Thioredoxins are small enzymes characterised by two proximal cysteine residues in the active site (CXXC motif). The free sulfhydryl groups (-SH) are oxidised to a disulphide bond (-SS-), and the electrons are transferred to acceptor molecules in the cells, RS, oxidised proteins and lipids, redox-signalling effector proteins, in a process of reversible oxidation. Reduction of thioredoxin is mediated by thioredoxin reductase (ThxRed), using NADPH as substrate [42,43]. Glutathione (GSH) is the primary antioxidant involved in redox state homeostasis and drug detoxification, serving as electron-donor in redox reactions. The two free sulfhydryl groups of two reduced GSH molecules are oxidised to form a disulphide bond, in the dimeric glutathione disulphide (GSSG). Through GSH oxidation to GSSG, Glutathione S-transferases mediate detoxification of cytotoxic substances and xenobiotics, while Glutathione Peroxidase (GPx) converts hydrogen superoxide to water and oxygen [44]. Glutathione reductase reconverts GSSG to GSH, using NADPH [45]; the GSH/GSSG ratio is a key biomarker of the cellular oxidative state.

## 2. Redox Signalling in Pregnancy

Pregnant women are exposed to increased levels of Reactive Oxygen and Nitrogen species (RONS) throughout pregnancy. Elevated levels of free radicals are crucial for the development of a successful foetal–maternal interface within the placenta making them indispensable for signalling transduction, activating proangiogenic genes, promoting vasodilation, and anti-inflammatory processes as well as activation of redox-sensitive transcription factors and protein kinases [46,47,48]. Activation of redox-sensitive transcription factors such as activator protein 1(AP-1), p53 and nuclear factor kappa-light-chain-enhancer of activated B cells (NF-κB) are essential for regulation of pro-inflammatory cytokines, cell differentiation, and apoptosis. Activation of protein kinases regulates the cell cycle and signalling pathways through regulation of mitogen activated protein kinase (MAPK).

**Figure 1:**
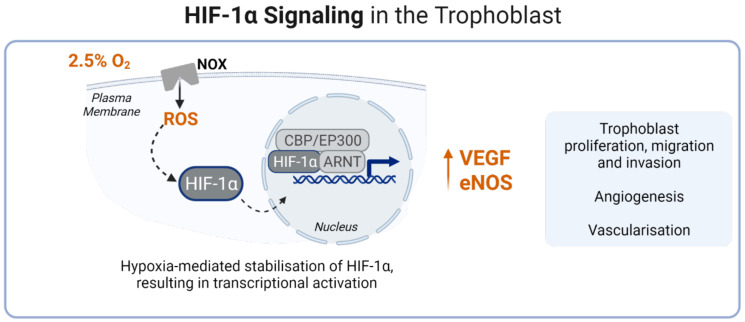
HIF-1α signalling in the trophoblast, during the first trimester. Dashed arrows indicate activation, upright arrow indicates increase, blue arrow indicates transcription, black arrow indicates generation.

RONS production occurs mainly in the placental mitochondria [48,49] During the early stages of pregnancy, low oxygen tension (2.5% O_2_) is a key activator and regulator of hypoxia-inducible factors (HIF-1α and HIF-2α) within the placenta, increasing the level of eNOS in extravillous trophoblasts and also modulation of the angiogenic mediator, vascular endothelial growth factor (VEGF). Increased levels of eNOS and VEGF stimulate invasion, angiogenesis, proliferation, and migration of trophoblasts into the maternal decidua [50,51,52]. Figure 1 recapitulates HIF-1α signalling in the trophoblast during the first trimester.

At the end of the first trimester, trophoblasts have invaded the maternal spiral arteries reaching the myometrium, which allows adequate blood flow from the mother to the placenta. Blood availability increases oxygen tension to 8.5% which promotes trophoblast differentiation and maturation. In syncytiotrophoblasts, increased oxygen levels coupled with demanding nutritional requirements stimulate mitochondrial metabolic activity leading to a switch in steroidogenic state, generating elevated concentrations of ROS [49,52,53]. In order to inactivate these free radicals, the placenta produces numerous antioxidant enzymes including CAT, SOD and GPx [52]. In addition, low-molecular-weight compounds, such as vitamin C, β-carotene, ascorbic acid, and glutathione, are produced by the placenta to combat rising OS [47,52].

Disruption of the delicate balance between antioxidants and oxidants increases free radicals in the maternal circulation and causes lipid peroxidation, protein degradation and ultimately cell death and ED [47]. Deficiency in the antioxidant defence, abnormal phosphorylation of the aforementioned transcription factors and protein kinases, results in an increase in soluble fms-like tyrosine kinase-1 (sFlt-1) levels and subsequent placental malperfusion [47,48]. Within the placenta, inefficient invasion of trophoblasts into maternal spiral arteries results in deficient blood flow, hypoxia, and ischemia, exacerbating free radicals release in the maternal circulation. Trophoblast damage results in the secretion of molecules implicated in ED, such as metalloproteases (MMPs), sFLT1 and soluble endoglin (sEng), inflammatory cytokines (TNF-α, IL-6), intracellular adhesion molecules (ICAM-1) and vascular cell adhesion molecules (VCAM-1) responsible for vascular remodelling, smooth muscle hypertrophy and cellular apoptosis [47,54].

The placenta, being a highly metabolic organ, requires a substantial amount of energy to maintain physiological and morphological functions and to support the growth and development of the foetus. Hence, the placenta has significant oxygen requirements throughout pregnancy [53]. Dysregulation of ROS signalling during pregnancy is linked to several pregnancy pathologies such as recurrent miscarriage, PE, IUGR, Gestational Diabetes Mellitus (GDM) and preterm birth [47,49,50,52].

**Figure 2:**
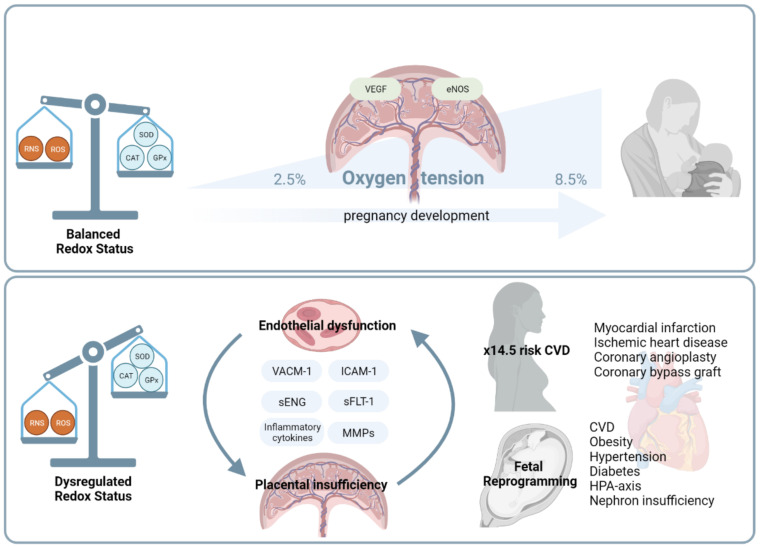
Role of Oxidative Stress in Pregnancy Pathologies.

The pathophysiological mechanisms involved in redox regulation during pregnancy are summarised in Figure 2.

### 2.1. Role of Oxidative Stress in Adverese Pregnancy Outcomes

Despite extensive investigation, a defined pathological relationship between OS and adverse pregnancy outcomes is yet to be fully elucidated. During GDM, the mother develops glucose intolerance in the second part of pregnancy. ROS associated with GDM is mainly produced by prolonged exposure to hyperglycemia. Glucose auto-oxidation increases the concentration of free radicals overwhelming the antioxidant response and resulting in increased protein carbonylation in the placental cell membranes [55]. Additionally, oxidative and nitrosative stress can exert synergistic effects. S-nitrosylation-mediated modifications result in structural alterations in term GDM placentas that show increased villous immaturity, increased angiogenesis and altered trophoblast proliferation and differentiation [56,57].

IUGR is characterized by the inability of the foetus to meet a standard growth curve. It has been hypothesized that the development of this pathology is due to first-trimester insufficient placentation and incomplete invasion of the maternal arteries leading to placental ischaemia [58]. Lipid peroxidation, DNA damage and ROS biomarkers (malondialdehyde, xanthine oxidase, glutathione peroxidase, superoxide dismutase and catalases) are elevated in pregnancies affected by IUGR [55].

Although there are several hypotheses on the aetiology of PE, the most common event is an injury to the vascular endothelium [58]. Increased vascular resistance reduces trophoblastic invasion of the maternal spiral arteries, leading to intermittent placental perfusion which increases ROS within the trophoblast and endothelial cells [55,58]. Other studies suggest the inability of the syncytiotrophoblasts to sense and generate ROS, possibly due to the abnormal regulation of NADPH in this cellular layer [55]. PE shows an extensive disturbance level of the maternal vasculature, which is not observed in IUGR pregnancies, but is present when PE and IUGR occur simultaneously. A recent theory for the development of PE has been attributed to poor maternal cardiovascular health failing to compensate for haemodynamic and metabolic requirement during pregnancy, a result of genetic or environmental risk factors [9,59].

In all the mentioned pregnancy pathologies, the placenta is in an environment of elevated concentrations of RONS, which may lead to the shortening of the telomeres and cellular metabolic arrest and induce cell senescence or promote placental apoptosis [55]. Thus, premature placental ageing of the foetal–maternal interface is exacerbated in the high oxidative environment and is commonly observed in adverse pregnancy outcomes.

### 2.2. Effects on Oxidative Damage to Foetal Health

Pathological pregnancies compromise the intrauterine environment, which may lead to foetal cells reprogramming to overcome the stressors. Foetal programming implicates alterations of physiologic systems of cardiovascular control, ultimately increasing the risk of future susceptibility to cardiometabolic diseases. This phenomenon is denominated the Developmental Origins of Health and Diseases (DOHaD), stated by Dr Barker after studying the association of maternal undernutrition with the increased incidence of coronary heart disease, hypertension, and obesity in adult life [60,61].

The most studied reprogramming association is the link between foetal undernutrition and the development of cardiovascular diseases and associated risk factors such as hypertension, cardiac hypertrophy, and obesity. However, other less studied associations have been observed. Increased levels of maternal cortisol are associated with behavioural problems during childhood. Excessive concentrations of glucocorticoids in GDM or obese mothers are associated with the reprogramming of the foetal hypothalamus-pituitary-adrenal (HPA)-axis at both the basal and the stress-induced level. OS is associated with the development of autism spectrum disorders, hypertension, heart diseases, a decreased number of nephrons and kidney dysfunction through the dysregulation of the Renin-Angiotensin system. In animal studies, intrauterine hypoxia induces foetal growth restriction, cardiovascular dysfunction, multiorgan morbidities associated with brain, heart, liver, and kidney [62,63,64,65]. Animal models have observed that hypoxia during intrauterine life induces left ventricular hypertrophy, susceptibility to develop arrhythmias and ischemia-perfusion injury. Undernutrition during the foetal life in sheep is linked to cardiac hypertrophy with the increased NADPH oxidase in the heart prior to the observation of hypertension [60].

Interestingly, foetal outcomes after intrauterine ROS imbalance have a sexual–dimorphic response. The female foetus seems to have a certain degree of protection, leading to reduced hypertension or a milder hypertensive form. Antioxidant deficiency equally seems to show the same sexual-dimorphism pattern, where female offspring express much higher amounts of renal antioxidants than male foetuses. This sexual dimorphic response can be explained by the prospective role of oestrogen’s ability to overcome and adapt to suboptimal intrauterine conditions in female foetuses [60,66,67,68].

### 2.3. Effects on Oxidative Damage in Pregnancy on Future Maternal Health

In healthy pregnant women there is systemic maternal vasodilation with a decrease in peripheral vascular resistance, increase in cardiac output, and thickness of the left ventricular wall. In pathological pregnancies, oxidative dysregulation and high concentrations of inflammatory cytokines increase ED, arterial stiffness, vascular resistance and impairs myocardial contractility, leading to left ventricular hypertrophy in the mothers. Persistent ED increases to a 14.5-fold risk of developing a poor cardiometabolic after delivery and years after delivery. PE is associated with a 2- to 7-fold increase in the risk of CV diseases later in life; 3% of PE women develop cardiopulmonary complications and 70% of these occur after delivery [69]. Women suffering from PE show subclinical atherosclerosis and are more likely to be treated for primary hypertension within 10 years after delivery. These associations are still significant after adjusting for confounding conditions before pregnancy, making PE a risk factor for the development of CVD and not only a cause of previous CVD [70,71]. It has been well studied that even 15 years after a pathological pregnancy, women still show changes in the microvascular function (cerebral microvascular bed), at the macrovascular level (increased arterial stiffness) and the structural vessel level (thicker carotid intima-media) [70,72,73]. Women suffering from PE have an increased risk of developing ischemic heart disease, myocardial infarction, coronary angioplasty, and coronary bypass graft [70].

Additionally, comorbidities such as insulin resistance, visceral obesity, increased high-density lipoprotein (HDL) cholesterol concentrations, and increased triglyceride concentrations accompanied by hypertension can lead to more deleterious effects on ED, leading to severe clinical outcomes such as CVDs and type II diabetes [54].

### 2.4. Oxidative Stress and Endothelial Dysfunction

The endothelium is a highly active monolayer of endothelial cells that line the interior surface of blood and lymphatic vessels and plays an important role in homeostasis and modulating immune inflammatory reactions. Endothelial cells regulate vascular tone by secreting vasoactive molecules responsible for relaxation or vessel constriction. This directly affects tissue oxygen supply, long-term organ perfusion and vascular structure remodelling [74,75].

**Figure 3:**
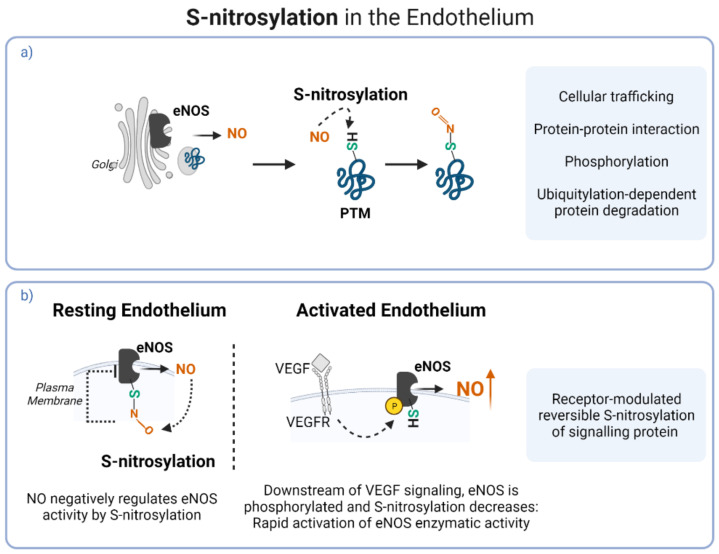
S-nitrosylation mechanisms in the endothelium. (**a**) eNOS-mediated S-nitrosylation of proteins as PTM. (**b**) S-nitrosylation negative, reversible, regulation of eNOS activity.

Under physiological conditions, endothelial cells generate vasoprotective NO to maintain the balance between ROS and antioxidants. NO signalling depends on eNOS cellular localisation. eNOS-mediated NO synthesis at the Golgi membrane causes S-nitrosylation of newly synthesised proteins, affecting cellular trafficking, protein–protein interaction and other PTMs processes (Figure 3a) [76,77]. At the plasma membrane, eNOS modulates vasodilation through NO synthesis. In resting endothelial cells, eNOS activity is reversible and is negatively regulated by NO itself through S-nitrosylation. Upon receptor-mediated activation of eNOS, such as downstream of VEGF signalling, eNOS is phosphorylated and S-nitrosylation levels decrease, resulting in strong activation of eNOS and dramatic increase in NO levels (Figure 3b) [78].

ED is defined by a decrease in the production of protective NO driven by elevated superoxide anion levels [79] and is one of the main risk factors for stroke and heart failure, while being a signature of PE. In general, ED is a state caused by an imbalance of vasodilation and vasoconstriction molecules [80], causing a disruption in the NO signalling pathway and leading to NO synthase uncoupling. eNOS and iNOS are constitutively expressed in the heart. In addition to superoxide anions, uncoupled NOS activity leads to peroxynitrite, which has been associated with aging in mice [81]. Likewise, several oxidative enzymes are involved in this process, and it has been shown that the inhibition of the NADPH oxidase normalizes ED in mice [82].

### 2.5. Oxidative Stress, NOX, and Cardiovascular Disease

The main sources of ROS in the context of CVD include NOX, xanthine oxidase, NO synthases, cytochrome P450, and mitochondrial respiration [83,84,85]. In the cardiovascular system, NOX1, 2, 4 and 5 are mainly expressed in certain cell types. For instance, NOX2 and 4 are expressed in cardiomyocytes, fibroblasts, endothelial cells, or smooth muscle cells, while NOX1, 4 and 5 are predominantly expressed in smooth muscle cells. NOX are generally regulated by Angiotensin II, which has both pro-hypertrophic and pro-fibrotic mediated effects in cardiac cells, notably via the endothelin-1 release [86].

Serpillon et al. (2009) showed elevated levels of superoxide in a rat model of type 2 diabetes was associated with endothelial but not cardiac dysfunction [87]. Additional studies have demonstrated that overexpression of NOX2 and 4 is associated with OS in CVD. In NOX4 knockout mice, decreased levels of superoxide were observed, while opposingly overexpression of NOX4 worsened cardiac function and induced apoptosis and fibrosis in response to pressure overload [88]. Both studies suggest NOX to be a potent source of superoxide in cardiac cells. Furthermore, patients with metabolic syndrome have increased NOX activity as well as elevated plasma levels of oxidized low-density lipoprotein and nitrotyrosine [89]. Another study in 2002 showed a correlation between NOX mRNA expression and the severity of atherosclerotic lesions in human coronary arteries [90]. Through these studies, the importance of NOX in CVD has been well established.

### 2.6. Therapeutic Interventions Targeting Oxidative Stress during Pregnancy

In addition to the physiological mechanisms previously mentioned, to prevent and repair damage from OS, other key regulatory molecules have been identified with clinical potential: enzymes such as ceruloplasmin, heme proteins, paraoxonase (PON1) or nonenzymatic albumin, bilirubin, uric acid, creatinine, cysteine, carotenoids, flavonoids, coenzyme Q (reduced), metal ion binding proteins (ferritin, transferrin, metallothioneins) [49].

Additionally, genetic, and environmental factors such as maternal obesity and poor nutrition may lead to increased OS during pregnancy. In this section, we describe the range of micronutrients, experimentally and/or clinically evaluated, as possible antioxidant therapies due to their ability to inhibit ROS production, scavenge existing ROS, regulate NO synthesis or release and modify antioxidant enzymes [91].

**Figure 4:**
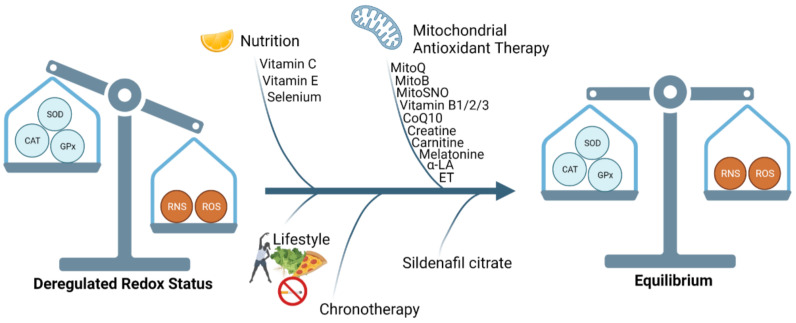
Interventions for Redox imbalance. Nutrition, lifestyle changes, chronotherapy, mitochondrial antioxidant therapy and sildenafil citrate interventions have been proven beneficial or are under investigation. alpha-LA = α-Lipoic Acid; ET = l-Ergothioneine.

An overview is given in Figure 4.

#### 2.6.1. Vitamins

Vitamin C and E are known exogenous antioxidants that regulate ROS through down-regulation of NADPH and upregulation of eNOS. Vitamin E protects cells against lipid peroxidation [92]. Additionally, Vitamin D supplements given in the early stage of pregnancy have been shown effective against PE through mediating immune modulation and vascular functioning [93]. However, most large-scale clinical trials with thousands of study participants have been inconclusive, rendering vitamins an ineffective treatment to reduce OS [92,94].

#### 2.6.2. Selenium

Selenium (Se), a trace element of amino acid selenocysteine, is mainly obtained from dietary components and is a vital element for GPx and ThxRed enzymatic function. Se deficiency during pregnancy can result in maternal and foetal morbidity and pregnancy complications such as intrauterine growth restriction (IUGR), gestational hypertension, and preterm birth. Various studies with Se dosage ranging from 60 to 200 μg/day intake between 12–24 weeks of pregnancy have been documented. However, recommendations for Se supplementation are currently not possible due to limited accuracy for Se homeostasis measurements and the poor understanding of complex interactions between micronutrients to different doses of Se [95,96].

#### 2.6.3. Lifestyle Intervention

Lifestyle modifications including regular exercise, weight loss, dietary changes, reducing toxic substances such as tobacco, alcohol and pollutants can help reduce OS and increase physiological antioxidant defence. Systematic reviews analysing the impact of physical exercise during pregnancy have concluded that exercise has a protective effect against PE [97]. Whereas other studies have contradictory advice on aerobic exercise, especially during early pregnancy [94]. Due to the heterogeneity in study results, and the lack of consensus on the optimal intensity of physical activity, its protective effects against OS in pregnancy pathologies cannot be satisfactorily assessed. Larger studies with well-defined methodological designs are required to strengthen the evidence that physical activity helps scavenge ROS and reduce OS.

#### 2.6.4. Sildenafil Citrate

NO has become a potential therapeutic approach for vasodilation of the fetoplacental circulation, embryo development and foetus growth [98]. NO activates soluble guanylyl cyclase to convert guanosine triphosphate (GTP) to cyclic guanosine monophosphate (cGMP), which is then degraded by phosphodiesterase 5 (PDE5) to guanosine monophosphate (GMP). Sildenafil citrate is a selective inhibitor of PDE5 and enhances the relaxation and cGMP accumulation. In a rat model, Sildenafil was shown to restore normal values of blood pressure, reduce proteinuria and plasma levels of sFlt-1 and sEng in preeclamptic rats. However, clinical trials showed no significant difference between control and preeclamptic women, while the Dutch arm of the multicentre STRIDER trial for the treatment of severe IUGR was halted due to detrimental effects of Sildenafil citrate on newborn survival [98,99,100].

#### 2.6.5. Chronotherapy

Circadian rhythms have a profound effect on human physiology and healthy cellular function. At a molecular level, the circadian clock is comprised of core transcription factors: CLOCK, BMAL1, Period (Per), and Cryptochrome (Cry), that act in a Transcription Translation Oscillating (TTO) loop [101]. Recent studies have shown that these factors play a role in the maintenance of blood pressure and metabolism. In an in vivo experimental model, BMAL1 knockout mice exhibited elevated endothelial damage, disrupted NO signalling and high ROS production, resulting in elevated oxidative damage [101,102]. In a 2013 randomised control trial in pregnant women, the group receiving chronotherapy in addition to low-dose acetylsalicylic acid (ASA) (100 mg/d) at 16 weeks, resulted in lower ROS-induced molecular damage, along with improved antioxidant enzymatic activity [103].

Melatonin, an antioxidant secreted by the pineal gland, is responsible for the maintenance of circadian rhythm and seasonal timing cues. In uncomplicated pregnancies, melatonin levels remain elevated during pregnancy and are responsible for growth and development of the foetal brain. However, in pregnancy complications such as PE, maternal circulating melatonin levels remain significantly low [104]. An in vitro and open label clinical trial study by Hobson et al. (2018), have shown that melatonin did not reduce OS or the production of the anti-angiogenic factors sFlt1, sEng, and activin A in preeclamptic placental explants. They concluded that while melatonin can be used as an adjunct therapy, it is not an efficient preventive therapy for ROS-induced pregnancy disorders [105].

#### 2.6.6. Mitochondrial Antioxidant Therapy

Mitochondrial therapy in the form of naturally occurring antioxidants and commercial drugs has been considered as a potential intervention for pregnancy disorders resulting from elevated OS.

Mitochondrial-targeted antioxidants include MitoQ [106], MitoB [107,108], MitoPerox, MitoSNO [109], and MitoTempo [110], which have been shown to decrease oxidative damage and ameliorating effects in ischemia-reperfusion injury [106] and cardiac hypertrophy [111].

Primary mitochondrial disorders in which oxidative phosphorylation is disrupted can further be treated with antioxidant nutritional interventions [112,113]. Vitamin B1 supplementation is commonly used to enhance oxidative phosphorylation system (OXPHOS) and pyruvate dehydrogenase complex flux. In patients with defects in riboflavin transport and processing, as well as electron transport chain (ETC) complex deficiencies, vitamin B2 supplementation has also shown similar positive effects [114,115,116]. Increased amounts of vitamin B3, a precursor of NAD^+^ and NADP^+^, can cause lactic acidosis, which can be mitigated by exercise and fasting [117].

Mitochondrial activity and biogenesis can be further modified by metabolic agents, such as coenzyme Q10 (CoQ10), creatine, α-Lipoic Acid (α-LA), and l-carnitine. CoQ10 acts as a diffusible electron carrier, transferring electrons from complex I and II to complex III of the ETC. CoQ10 further inhibits the propagation of lipid peroxidation and is a potent ROS scavenger [118]. Creatine supplementation has been shown to prevent both structural and functional damage to mitochondria of cells under OS [119,120]. The potent antioxidant α-LA has been shown to improve mitochondrial performance and epigenetically affect IL-1B and IL-6 gene expression, which are associated with OS and inflammation. However, its therapeutic benefit has yet to be supported by clinical trials [121,122].

l-carnitine is involved in long-chain fatty acid transport from the cytosol to the mitochondrial matrix and is important for β-oxidation, modulation of acyl-CoA/CoA ratio, excretion of toxic acyl groups, muscle storage of energy as acetyl-carnitine and additionally functions as a free radical scavenger [123].

Finally, l-Ergothioneine (ET) is a histidine derived dietary amino acid and known for its radical scavenging properties, demonstrating reduction in markers of OS in serologic samples taken from healthy human subjects, who were administered ET (5 or 25 mg) once a day for 7 days [124,125]. Using the reduced uterine placental perfusion (RUPP) rat model, Williamson et al. (2020) [126] showed that ET treatment significantly ameliorated hypertension, increasing pup weight, decreasing circulating levels of antiangiogenic sFlt-1 and significantly reducing mitochondria-specific H_2_O_2_ in vivo. ET has a favourable safety profile, long half-life, cytoprotective properties, and has the ability to regulate mitochondrial function; hence, ET could become a potential therapy for ROS-induced pregnancy disorders such as PE and IUGR [126].

Nevertheless, a considerable amount of research is still needed to accurately understand the exact processes involved in the metabolism of these therapeutic interventions.

The mechanisms by which hypoxia can lead to a higher incidence of cardiovascular dysfunction in the offspring are not fully understood; however, evidence suggests that prenatal hypoxia is linked to abnormal development of the foetus including the cardiovascular system. A study in a rat model of maternal malnourishment showed that early postnatal supplementation with Coenzyme Q10 had beneficial effects on the developmental origins of CVD, reducing signs of cardiac aging, telomere shortening and cellular senescence [127]. Another study in a mice model demonstrated that prenatal hypoxia leads to sex- and age-dependent effects on offspring cardiac and vascular function later in life. MitoQ may have potential benefits in offsetting the cardiovascular pathologies by increasing sensitivity to vasorelaxation in aged male and female offspring, preventing pulmonary artery dysfunction in prenatally hypoxic young male offspring and improving systolic function in aged prenatally hypoxic female offspring [128].

While limited to in vivo models, these potential therapeutic benefits are encouraging and warrant further exploration of mitochondrial antioxidants as an intervention to prevent long-term negative cardiovascular outcomes of a suboptimal in-utero environment.

### 2.7. Detection and Assessment of Oxidative Stress

Biomarkers for OS and oxidative damage are exploited as tools to assess both the status of a human disease and health enhancing effects of antioxidants.

#### 2.7.1. Measurement of RONS

ROS and RNS are the key molecules of OS [129]. Direct measurements of their cellular distribution are a common approach to determine OS severity. Most common methods consist of colorimetric and fluorometric assays. Cellular RONS levels can be estimated using fluorogenic probes, such as Dichlordihydrofluorescein-diacetate, which is reduced to dichlorofluorescein by ROS, changing its fluorescent properties [130]. Similarly, 4,5-diaminofluorescein (DAF-2) is subject to nitrosation in the presence of RNS, forming the highly fluorescent triazole DAF-2 T [131]. Dihydroethidium is a viable cell permeable probe, which produces fluorescence upon oxidation by RONS and thus a measure for RONS activity [132]. These probes lack specificity and better approximations can be achieved by combining these measurements with specific inhibitors of RONS synthases as well as scavengers [133].

Colorimetric assays apply to a wide range of RONS. Most H_2_O_2_ specific dyes, such as Amplex^®^ Red [134], homovanillic acid [135], and tetramethylbenzidine [136], function in combination with Horseradish peroxidase (HRP). A general limitation is due to HRP reacting with other thiols, possibly skewing the result; therefore, it is suggested for H_2_O_2_ detection in cultured cells, organ cultures, and isolated buffer perfused tissues [137]. To distinguish cellular from mitochondrial ROS, additional mitochondria specific dyes, such as MitoSOX™ and CellROX ^®^ can be applied [138,139]. Another approach is the measurement of ROS derivatives. Transition metal ions (Fe^2+^, Fe^3+^) form radicals by reacting with hydroperoxides; these radicals can be measured colorimetric and are proposed to be proportional to the amount of peroxides present in the sample [140].

A historical colorimetric assay for RNS detection is the Griess reaction (1879) based on the interaction of nitrite, under acidic conditions, with a series of organic compounds (i.e., sulfanilic acid, 1-naphthylamine) to produce a red-violet dye. This is measured by spectrophotometry with a limited sensitivity (500 nmol/L), improved by fluorometric detection [133,140].

NO concentrations can further be assessed based on their effects on vasodilation. In this bioassay, endothelial cells are grown on carrier beads that fill a capillary column linked to a fluidic system. In the downstream chambers, the tension of smooth muscle cells from aortic vessels, stripped from the endothelium, is assessed by isometric transducers. Muscle relaxation is proportional to NO levels synthesised by the endothelium [29,141]. This method has been extensively used to characterise NO-mediated regulation of vascular function in response to hormones and bioactive molecules, and have been further developed to allow for modulation of oxygen tension [142].

#### 2.7.2. Assessment of Oxidative Damage

Despite the variety of fluorescent probes described above, ROS assessments remain challenging. These species are by definition highly reactive and possess a short lifespan. Examining the damage caused by OS to proteins, lipids, and nucleic acids is an alternative approach.

Protein Damage: ROS oxidizes amino acid residues and protein backbones, increasing protein carbonylation and nitrosylation levels, serving as a marker of OS. The global extent of these modifications on the proteome can be measured using 2,4-dinitrophenylhydrazine, 2D gel electrophoresis, Western blot, and OxyBlot [143,144].

Lipid Damage: Lipid peroxidation is widely used as an indicator of free radical formation. Membrane unsaturated fatty acids are targeted by free radicals, creating peroxy radicals. Traditionally, lipid peroxidation is measured by thiobarbituric reactive compound detection, which is generated from the reduction of lipid peroxidation products. Although this method is highly sensitive, it lacks specificity.

High specificity and direct quantitative measurement of lipid peroxidation products can be achieved with chromatography techniques, including High-Performance Liquid Chromatography (HPLC) and Gas Chromatography-Mass Spectrometry (GC-MS) [145,146].

Compared to other liquid chromatography techniques, in HPLC, the high-pressure pump supplying the solvent and the small column calibre grant faster separation of lipid species at a higher resolution [147]. HPLC alone is the gold standard for quantification of known biomarkers such as Malondialdehyde (MDA) and 4-hydroxy-2-nonenal (4-HNE), commonly used in clinical and functional studies [148,149].

In GC-MS, GC is used to separate small volatile or semi-volatile compounds that can withstand high-temperatures without degradation, such as fatty acids [150]. Then, MS allows the precise identification of peroxidation products, establishing the position of hydroxyl groups along the lipid chain. Although it is expensive and time-consuming, MS is the technique of choice for untargeted lipidomics [151,152].

DNA Damage: The most prominent oxidative modifications of nucleic acids are hydrogylation of deoxyguanosine residues, thymidine glycol, single and double-stranded breaks, and levels of DNA repair enzymes. Specifically, 8-Hydroxy-2′-deoxyguanosine is extracted by the DNA repair system and then circulates in both the blood and urine. Common methods for quantitative analysis are HPLC, GC-MS, tandem HPLC and Elisa [153,154]. HPLC and immunohistochemistry (IHC) can also be used to assess DNA repair enzyme levels.

To assess DNA strand breaks, polymerase chain reaction and agarose gel electrophoresis exploit the fact that cleaved DNA fragments can migrate out of the nucleus and migrate faster than undamaged DNA when exposed to an electric field. Single-cell gel electrophoresis, also known as a comet assay, is considered the gold standard [155].

In vivo measurements remain challenging. The analysis of oxidized lipids in vivo is limited by their low abundance and the diversity of previously described physicochemical properties.

However, most experimentally identified therapeutic targets do not pass clinical trials. This can be due to a negative outcome of a clinical trial, due to safety or efficacy issues and clinical trial failure due to expertise-based, execution based, or knowledge-based failure [156]. Hwang et al. reported that 54% of therapeutics failed during clinical development, of which 57% failed due to inadequate efficacy, 17 due to safety concerns and 22% due to commercial reasons [157]. The investigation of therapeutics for pregnancy complications is particularly challenged by the fact that pregnant women are often excluded from clinical trials. Trials that are based on a different population can lead to approval of therapeutics that behave unexpectedly, even negatively, in the actual target population [158].

A detailed collection of OS assessment methods can be found in Supplementary Materials S1.

## 3. Current Computational Models—Enhanced Interpretation of Complexities of Oxidative Stress

Complex interactions and high throughput data integration are often modelled computationally to simulate and study mechanisms leading to a certain phenotype. This allows the performance of thousands of in silico experiments that have to be validated by only a limited amount of laboratory experiments. Both predictive and quantitative computational models of redox reactions have been created to enlighten the mechanistic features of redox-sensitive proteins [159].

Computational models are usually based on experimental data and high-throughput methods and represent the current state of knowledge. However, the modelling process and in silico experiments can further be used to test hypotheses, interpret the results of in vitro and in vivo experiments, refine experimental designs, and lead to new insights [160].

Static models are used to describe the relationships between model components, while dynamic models are primarily used to describe changes over time. Protein–protein interaction networks are a popular example of static models. However, components, such as proteins, can also be described in dynamic models, using, i.e., ordinary differential equations (ODE) describing how components interact with each other, and how interactions are changed in response to stimuli. ODEs are used in deterministic models that, by definition, give the same output every time.

**Figure 5:**
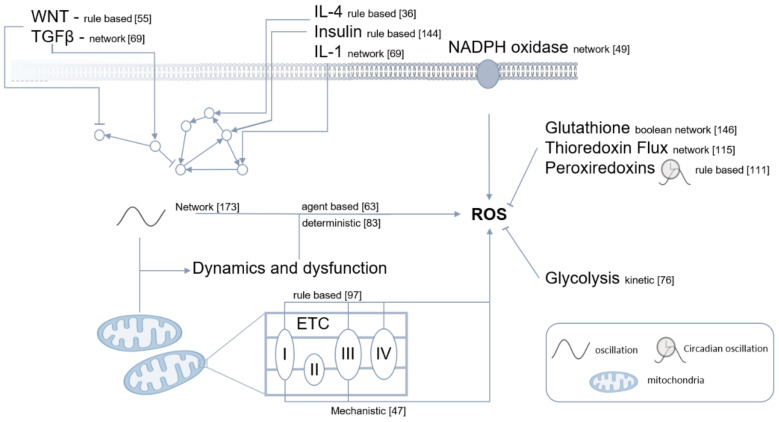
Interacting redox-sensitive pathways with existing computational models. The generalized model type is named, including a model reference. Detailed information can be found in Supplementary Material S2.

Notwithstanding, biological systems contain a certain amount of uncertainty. Stochastic simulations allow the modelling of systems containing rare, random, and uncertain reactions. As these simulations vary between runs, they must be repeated until a conclusive result is reached. Stochastic models include algorithms, such as Monte Carlo, Markov chain, Random walk [161]. Random protein damage via ROS modelling benefits from stochastic algorithm use [162]. The following paragraphs discuss the latest redox specific models, grouped by model type (Figure 5).

Computational models are usually created based on a specific hypothesis. Although the idea of a comprehensive model is appealing, the computational cost under current conditions would be too high and thus difficult to realize. Models are usually used to describe behaviour and mechanisms resulting in a specific behaviour. As mitochondria are the main producers of ROS, most redox-specific models focus on mitochondrial function. The model by Gauthier et al. (2013) revealed how ROS levels are controlled by the redox balance in mitochondria, which was later extended to show mitochondrial complex 1 as the main ROS producing site. This information is beneficial in drug development and treatment decision making [163,164]. Other models revealed the specific interplay of redox-sensitive proteins [165]. The inclusion of competing redox mechanisms revealed reversible protein tyrosine phosphatase oxygenation via ROS as the primary regulator in IL-4 signalling. This result has implications in therapeutic methods for ROS-related immune response deregulations [166].

More complex approaches, such as multimethod multicell simulations, including stochastic damage-dependent frequencies, illuminated mitochondrial dynamics and dysfunction specific to ageing [167]. Even models of the oscillatory relationship between ROS and ROS scavengers [168], and the role of peroxiredoxins (Prxs) and silfiredoxin for cellular circadian redox oscillation [169], have been created.

The diversity of redox regulated processes and tissues in which they occur indicates that special effects may have to be considered. This increases the complexity of a model and changes its construction. To support more integrative approaches, instead of ODE, partial differential equations and agent-based models, in which different compartments can be considered, are applied. System biology approaches focus on network modelling, which is characterized by viewing cells and molecular processes within them on different levels. This approach consists of two methodologies: static large-scale biological network modelling focusing on omics data integration, visualization, and topological analysis; and dynamic quantitative modelling.

### Network-Based Redox Models

Mitochondrial function has also been described in network-based models, supporting the function of oscillating mitochondria in cardiomyocyte cell death [170]. Boolean network modelling of OS, set out to pinpoint dysfunctional components in OS responses using temporal variations, described the role of GSH in the cellular redox state and its role in ROS reduction and apoptosis inhibition [171]. Even the redox concentration dependency of insulin signalling has been studied using ODEs, which has implications for insulin-related disorders [172].

Computational models can not only describe existing mechanisms. An ideal example of how experimental and computer science benefit from one another is the Padayachee et al. (2020) thioredoxin model. The Thioredoxin system flux is an important indicator of the function and efficiency of the thioredoxin system within a biological sample. Flux has traditionally been measured in vitro by NADPH oxidation, while the thioredoxin redox state has been used as a proxy for the activity of the system. In vivo measurement of the flux, however, has remained challenging. This model suggested that in vivo thioredoxin oxidation measures can be used as surrogate indicators for flux, thus opening up new methods to experimental approaches that can lead to more accurate results [173]. Thiol state prediction models have yet to be adapted to animal systems [174]. Other computational redox models related to ageing have been extensively reviewed elsewhere [162,175].

Large networks can be turned into molecular interaction maps (MIM) as part of disease maps (DMs), allowing omics integration [176,177]. MIMs describe molecular interactions in a machine and human readable fashion and are frequently used for advanced network analyses [176,178]. DMs are a specialized approach for complex modelling, which supports the inclusion of compartments. Systems Biology Markup Language (SBML) is a machine-readable format that can be intuitively interpreted by users. SBML models consist of species, such as genes, miRNA, proteins, and other molecules, and biochemical reactions. Most system biology tools, such as CellDesigner, Newt, Copasi, and Cytoscape, are freely available and give introductions to biologists unfamiliar with computational modelling. The research area includes the objective to increase the reproducibility and reusability of models [179].

These techniques have not been fully exploited in the context of maternal and fetal health and there is currently no comprehensive redox-signalling model resource, which is not surprising considering the number of different processes and tissues redox signalling is involved in. However, there are a number of models related to specific redox-signalling pathways. Mitochondria-specific models have recently been reviewed [180]. Other resources include the PantherDB “oxidative stress response” pathway. Reactome further provides ROS specific pathways “oxidative stress induced senescence” (R-HSA-2559580), “cell redox homeostasis” (R-HSA-1222541), “detoxification of reactive oxygen species” (R-HSA-3299685), and “ROS and RNS production in phagocytes” (R-HSA-1222556), and the CardioGlyco model [181].

Other models worth mentioning in this review are biophysics quantum and atom level models of electron transfer. This enabled the accurate estimation of biological macromolecule redox potentials and stressed the importance of long-range electrostatic interactions [159], with the outlook to study the role of redox potentials on DNA [182]. Of particular interest were the electronic properties of the transition metals iron and zinc in proteins, as it has an effect on structure, function, and stability of the metalloproteins [183,184]. These models, however, cannot currently be integrated into more complex system models due to technical limitations. A detailed table of redox-related models can be viewed in Supplementary Materials S2.

## 4. Future Perspectives

Pregnancy-related research entails challenges. The inherent vulnerability of the patient groups, as well as common comorbidities in pregnancy complications, increase the required number of biological samples for statistical analysis significantly. The use of cell lines, explant cultures, animal models, and computational modelling are useful circumventions [160].

Redox signalling plays a major role in both successful and complicated pregnancies. We have shown the plethora of processes falling under the term “redox signalling” and associated maternal and foetal outcomes. The complexity of redox signalling requires consideration of more than one biomarker to obtain meaningful results. On the experimental side, this calls for more integrative approaches considering both pro- and antioxidant reactions with a higher sensitivity to physiological variation.

Although computational modelling of redox-related processes is often utilized in health sciences, these models have not been exploited in the context of feto-maternal health research, notwithstanding that computational modelling reduces the need for experimental replicates by identifying biomarker candidates [160].

**Figure 6:**
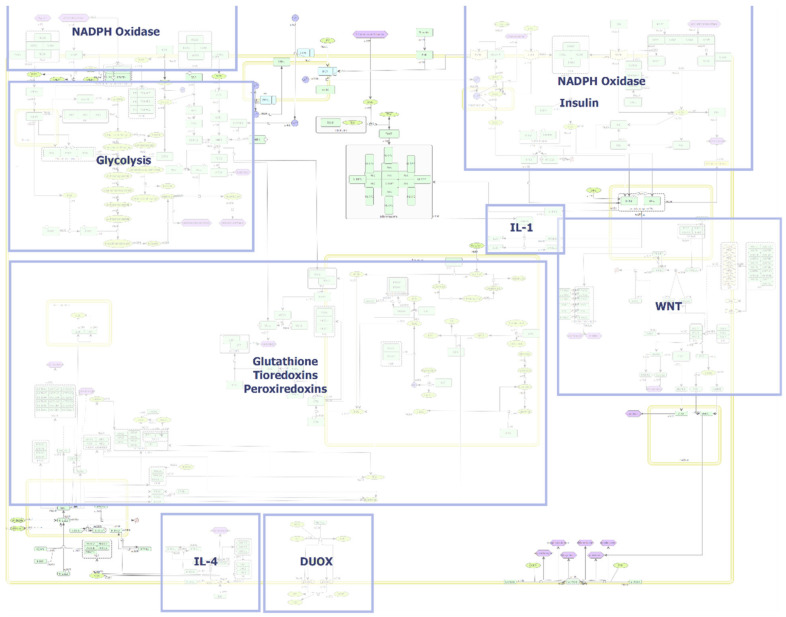
Redox signalling map grouped according to signalling process. Interacting species are indicated as nodes and their interactions as lines. Interactions can be “inhibition”, “positive influence”, “modulation”, “state transition”, and “transport”. Unclear interactions are visualized as dashed lines. (Green = proteins; bright green = simple molecules; purple = phenotypes; blue = ions).

The combination of existing models to a larger network, including other redox-signalling pathways, may lead to deeper insights to provide the opportunity to explore interventions and their effects on complex biological processes [185]. To this end, we created a redox signalling SBML model (Figure 6) available in Supplementary Materials S3, which will be integrated into the placenta-specific disease map currently under development.

This map includes proteins, chemical entities, receptors, and phenotypes involved in ROS, RSS, and RNS pathways and previously described redox-sensitive protein signalling. Notably, mitochondrial ROS production is not integrated here, as it has been previously created [180] and can be extended using the presented map. Both activity flow and progress description are included, increasing human interpretability. Activity flow describes the general flow of information between elements, while process descriptions include detailed mechanistic descriptions of molecular interactions [186]. The redox signalling map contains 224 unique proteins, 58 simple molecules, 238 interactions, and 5 compartments (general cell, nucleus, mitochondrion, endoplasmic reticulum, and peroxisome) making spatial considerations possible. The redox map was created in CellDesigner and is SBML compliant. Each species is annotated to enable database linkage. Proteins are named according to gene name conventions, simple molecules contain ChEBI naming conventions; all reactions are annotated with the respective publication they were extracted from.

## 5. Conclusions

In this review, we aimed to summarize the mechanism of redox signalling, briefly discussing the different reactive species affecting pregnancies and maternal health at molecular and physiological levels. We also discussed various therapeutic interventions investigated through multiple models and their potential benefits.

Despite ample evidence of the importance of the redox status in pregnancy pathologies, the exact molecular mechanism remains to be elucidated and determination of oxidative damage remains challenging, even with advanced research methodologies. A closer collaboration between experimental and computational scientists yields opportunities to alleviate both experimental challenges and the general challenge of recruiting samples from the vulnerable patient groups. The computational models described in this review can be used to unravel complex signalling pathways. With the combination of knowledge held within these models, we created a molecular interaction map of redox signalling processes. This model can be used in combination with pregnancy pathology specific data to elucidate the molecular interplay involved in the pathology of interest (Supplementary Materials S4). Henceforth, as hypothesised by Hoch et al. (2021), this approach enables the identification of signalling regulators and potential therapeutic targets [63].

## Supplementary Materials

The following supporting information can be downloaded at: https://www.mdpi.com/article/10.3390/antiox11030585/s1, Supplementary Materials S1: Table of experimental methods to assess redox status, Supplementary Materials S2: Table of computational models of redox sensitive pathways., Supplementary Materials S3: SBML compliant CellDesigner Model in xml format of redox signalling map, Supplementary Materials S4: Brief outlook on redox signalling map application using public data.
