# Supplementary material for: Computational Models on Pathological Redox Signalling Driven by Pregnancy: A Review
Source: Antioxidants (Basel). 2022 Mar 18;11(3):585. doi: 10.3390/antiox11030585 (PMC8945226; doi:10.3390/antiox11030585)
Supplement: Supplementary file 1 [file antioxidants-11-00585-s001.zip › Supplementary material 1.pdf]

Table of experimental methods to assess redox status.

Method/Main Component	Input or general Information	Output	Challenge	Reference
**ROS**				
Ferricytochrome c reduction to ferrocytochrome c	ferricytochrome c reduction to forrocytochrome c<br>Absorbance based	Indicator for ROS formation rate	Outdated - indirect + less sensitive compared to current methods	(19)
Lucigenin (redox cycling)<br>Coelenterazine	Chemilumenscent reactions to determine ROS production	Indicator for ROS formation	Lucinogen - not ideal for quantitative measures<br>Coelenterazine does not redox cycle - not specific to ROS alone - also to peroxnitrite	(10)
Hydrocyanine dyes	Fluorogenic sensors for ROS and hydroxyl radicals, functions both *in vitro* and *in vivo*	Indicator for ROS and hydroxyl radicals	High auto-oxidation<br>Low strokes shift<br>Low solubility	(16)
Dihydroethidium	Fluorescence until oxidized by ROS and RNS<br><br>Spectroscopy	Marker product of ROS and hydroethidine	Differentiation between ROS and RNS oxidative function not possible - only semi quantitative (if conditions are compared)	(36)
ROS	• redox-sensitive fluorophores<br>• fluorescent reporters<br>• electron paramagnetic resonance spectroscopy<br>• HPLC	ROS quantity		(32)
**Mitochondrial ROS**				
MitoSOX™ assay	Cationic derivative of dihydroethidium<br>- permeates live cells and targets mitochondria<br>- In live cells - fluorescence	Mitochondrial ROS	Cannot differentiate between different ROS species	(15)
CellROX ®	Cell-permeant dyes that are weakly fluorescent in reduced state and exhibit stable fluorescence upon oxidation by ROS	Dependent on specific CellROX type either mitochondrial ROS	Conflicts with H_2O_2 responses	(33)
Mitochondrial membrane potential indicator for OS	In vitro method<br>Usually coupled with oxygen consumption rate<br>Time-series TMRM fluorescence measurement	Indicator for ROS mediated cell injury		(31)
**H_2O_2 detection**				

Amplex ® Red	Amplex Red is oxidized by H_2O in presence of HRP and then converted to resorufin<br>Detection colorimetric or fluorescence	H_2O_2 detection	Highly sensitive to light exposure and then less sensitive to H_2O_2	(35)
Homovannilic acid	Oxidizes with H_2O_2 through HRP<br>Detection via fluorescence	H_2O_2 detection	Sensitive to background signal especially when polystyrene microplates are used	(23)
TMB	Oxidizes with H_2O_2 through HRP	H_2O_2 detection	Less sensitive, but cheaper<br>General drawback with HRP catalysis - thiols skew the result	(34)
H2DCF	Is reduced to DCF by H_2O_2 (and other ROS)<br>Detection via fluorescence	H_2O_2 detection	Originally used for H_2O_2 detection until it was shown not to be specific to H_2O_2<br><br>H_2O_2 specific extensions: Peroxy Green1 and Peroxy Crimson1	(20)
**Nitric Oxide**				
Nitrate and nitrtite<br><br>Follow up with nitrite only assay<br><br>"Griess assay"	NO_3 reduction to NO_2 by nitrate reductase. Nitrite forms nitrous acid in presence of hydrogen ions<br>- nitrous acid reacts with sulfanilamide and produces diazonium ion<br>Detection colorimetric	Indirect NO detection	Indirect and unspecific	(11)
NADH and NADPH	Detection via cellular autofluorescence + FLIM	NAD(P)H redox state (saparation of NADH and NADPH)		(3)
Fluorometric probe: DAF-2	DAF-2 reacts with either N_2O_3 or NO_2 to form highly-fluorescent triazole	Detection of NO and RNS	Susceptible to experimental artifacts (increased fluorescence by Ca^{2+} ions, incident light and non-neutral pH).<br>Cross reactivity with peroxynitrite, nitroxyl, and ascorbic acid	(13)
Fluorometric probe: DAN	DAN reacts with nitrous acide and forms 2,3 naphthotriazole<br>Detection via fluorescence<br>Works both *in vitro* and *in vivo*	Indicator of NO formation	Not able to inform about area of NO production	(4)
Bioassays cGMP level<br><br>Vessel relaxation	Details stated in method title	Measurement of downstream actions of NO	Indirect measurement<br>Do not adjust for other confounding factors	(21)

Inhibition of platelet aggregation				
**Glutathione**				
HPLC	Uses iodoacetic acid to modify thils and 1-fluoro-2,4-dinitrobenzene to add a chromophore to the amino groups, allowing direct measurement of GSH, GSSG, Cys, CySS, and CySSG	GSH to GSSG ratio	Not sensitive enough to measure GSSG in plasma	(14)
• Capillary electrophoresis<br>• Free zone CE<br>• Micellar electrokinetic chromatography<br>• Isotachophoresis<br>• Capillary gel electrophoresis<br>• electrochromatography	Electrophoretic separation by application of high electric field to sample in capillary - background electrolyte (BGE)	GSH to GSSG ratio	Limited analyte loading leads to diminished sensitivity	(6)
Microplates		GSH to GSSG ratio	Prone to human error	(2)
Luciferase assay (example Promega)	Luciferin derivative glutathione s-transferase - GSH amount proportional to luminescent signal when luciferase is added	GSH quantity	Prone to human error	(25)
Colorimetric assay:	GSH + DTNB + glutathione reductase	Reduced GSH quantity	Prone to human error	(12)
Monobromobimane	Monobromobimane and monochlorobimane react with low molecular weight thiols (incl glutathione) and form fluorescent adductcs	GSH ratio before and after chemical reaction	Monochlorobimane more thiol selective however, accumulates in the nucleus	(17)
ThiolTracker Violet	Reacts with reduced thiols in intact cells → follow up flow cytometry and fluorescence microscopy	Glutathione detection	Measures thiols in general, GSH is approximately measured	(18)
Lipid peroxidation				
MDA	Reaction of membrane unsaturated fatty acids with free radicals generates lipid peroxides - MDA and TBA adduct can be measured	TBA	Highly sensitive but not specific to MDA	(26)

IsoP Elisa	Stated in method title	IsoP quantity	Requires purification of samples	(22)
IsoP GC/MS<br>*In vivo*	Plasma or urinary samples<br>GC/MS	IsoP quantity	High cost	(28)
BODIPY®<br>Image-IT®	Live cells - fluorescent-Mass spectometry	IsoP ratio	Sensitive to photo bleaching in high-intensity illumination conditions	(8)
LAA Click-iT®<br>Alexa Fluor ®	LAA incorporates into cellular membrane and is oxidized in response to lipid peroxidation - LAA then produces HPODE that modifies surrounding proteins<br>- the resulting alkyne-containing proteins can be detected using AlexaFluor or Click-iT	Lipid peroxidation reporter	Cells must be fixed	(37)
• HPLC<br>• HPLC-MS	Used to separate lipid mixtures in fractions, based on molecular weight and polarity..<br><br>Gold standard for quantification of known biomarkers	• Lipid peroxidation biomarkers<br>• Specific identification of unknown peroxidation derivatives of large nonvolatile lipids	In isolation, requires known standard.<br><br>Quantification can be imprecise if the molecule of interest has similar molecular weight of other molecules in solution<br><br>High financial and time cost	(24)
• GC<br>• GC-MS	Used to separate lipid mixtures in fractions, based on their volatility.<br><br>Followed by MS allows identification of unknown peroxidation products	• Lipid peroxidation biomarkers<br>• Specific identification of unknown peroxidation derivatives	Requires high temperatures<br><br>Quantification can be imprecise if the molecule of interest has similar molecular weight of other molecules in solution<br><br>High financial and time cost	(9)
**Redox Status**				
Genetically encoded sensors	*In vivo*<br>Change fluorescence in response to alteration in redox state i.e. roGFP - linked to Orp1 which forms disulfides after reacting with H_2O_2	GSH redox potential	Monitoring redox changes in multiple compartments remains difficult	(27)
2D electrophoresis PAGE (DIGE)	Cysteine-labeling	Identification of redox sensitive proteins specific to user defined conditions	High cost	(7)

DNPH	DNPH reacts with all protein carbonyls	Protein carbonyl content	Interacts with heme groups	(1)
OxiBlot	• DNPH reacts with protein carbonyls<br>• Followed by separation of oxidatively modified proteins by elecrophoresis<br>• Followed by westernblot with anti-DNP antibodies	Protein carbonyl content	Time consuming	(29)
8-hydroxy-2' -deoxyguanosine	Detection methods:<br>• HPLC<br>• GC-MS<br>• HPLC tandem<br>• ELISA	Indicator for DNA damage caused by OS	High cost and low throughput	(30)
In vivo Measurements of Ca^{2+}, pH, redox state	• Genetically encoded fluorescent reporters<br>• Two photon microscopy<br>• nuclear magnetic resonance	• Ca^{2+}<br>• pH<br>• redox state	High expertise required<br>Specialized equipment required<br>High cost	(5)

Table of experimental methods to assess redox status, sorted by output. Included are the general name the method is referred to, as well as keywords describing the method and/or reactions the method is based on, outcome information, challenges and a reference referring to either a publication describing the method, or a product link. Methods that can be used for several biomarkers are only explained once.

**Abbreviations**			
DAF-2	: Diaminofluorescein	IsoP	: Isoprostane
DAN	: 2, 3-diaminonaphthalene	LAA	: Linoleamide alkyne
DNPH	: 2,4-dinitrophenylhydrazine	MDA	: Malondialdehyde
DTNB	: 5,5 dithio-bis-nitrobenzoic acid	N_2O_3	: Dinitrogen trioxide
GC/MS	: Gas chromatography–mass spectrometry	NADH	: Nicotinamide adenine dinucleotide hydrogen
GSH	: Glutathione	NADPH	: Nicotinamide Adenine Dinucleotide Phosphate Hydrogen
GSSH	: Oxidized glutathione	NO_2	: Nitrogen Dioxide
H2DCF	: 2,7-dichlorodihydrofluorescein diacetate	NO_3	: Nitrate
H_2O_2	: Hydrogenperoxyde	roGFP	: Reduction-oxidation sensitive green fluorescent protein
HPODE	: 3-hydroperoxyoctadecadienoic acid	TMB	: Tetramethylbenzidine

Bibliography:

1. Alomari, E, Bruno, S, Ronda, L, Paredi, G, Bettati, S, and Mozzarelli, A. Protein carbonylation detection methods: A comparison. Data Br. Elsevier, 2018;19:2215–20.
2. Baker, MA, Cerniglia, GJ, and Zaman, A. Microtiter plate assay for the measurement of glutathione and glutathione disulfide in large numbers of biological samples. Anal Biochem. 1990;190:360–5.
3. Blacker, TS, and Duchen, MR. Investigating mitochondrial redox state using NADH and NADPH autofluorescence. Free Radic Biol Med. Elsevier, 2016;100:53.
4. Bowman, LAH, Mclean, S, Poole, RK, and Fukuto, JM. The Diversity of Microbial Responses to Nitric Oxide and Agents of Nitrosative Stress: Close Cousins but Not Identical Twins. Adv Microb Physiol. 2011;59:135–219.
5. Breckwoldt, M, Pfister, F, Bradley, P, Marinković, P, Williams, P, Brill, M, et al. Multiparametric optical analysis of mitochondrial redox signals during neuronal physiology and pathology in vivo. Nat Med. Nat Med, 2014;20:555–60.
6. Camera, E, and Picardo, M. Analytical methods to investigate glutathione and related compounds in biological and pathological processes. J. Chromatogr. B Anal. Technol. Biomed. Life Sci. Elsevier, 2002.pp. 181–206.
7. Chan, HL, Sinclair, J, and Timms, JF. Proteomic analysis of redox-dependent changes using cysteine-labeling 2D DIGE. Methods Mol Biol. 2012;854:113–28.
8. Drummen, G, Gadella, B, Post, J, and Brouwers, J. Mass spectrometric characterization of the oxidation of the fluorescent lipid peroxidation reporter molecule C11-BODIPY(581/591). Free Radic Biol Med. Free Radic Biol Med, 2004;36:1635–44.
9. Fisk, HL, West, AL, Childs, CE, Burdge, GC, and Calder, PC. The Use of Gas Chromatography to Analyze Compositional Changes of Fatty Acids in Rat Liver Tissue during Pregnancy. J Vis Exp. MyJoVE Corporation, 2014;51445.
10. Freeman, BA, Tarpey, MM, White, CR, Suarez, E, Richardson, G, and Radi, R. Chemiluminescent Detection of Oxidants in Vascular Tissue: Lucigenin But Not. Circ Res Circ Res. 1999;
11. Green, L, Wagner, D, Glogowski, J, Skipper, P, Wishnok, J, and Tannenbaum, S. Analysis of nitrate, nitrite, and [15N]nitrate in biological fluids. Anal Biochem. Anal Biochem, 1982;126:131–8.
12. Hedley, DW. Flow Cytometric Assays of Anticancer Drug Resistance. Ann N Y Acad Sci. 1993;677:341–53.
13. Itoh, Y, Ma, FH, Hoshi, H, Oka, M, Noda, K, Ukai, Y, et al. Determination and Bioimaging Method for Nitric Oxide in Biological Specimens by Diaminofluorescein Fluorometry. Anal Biochem. Academic Press, 2000;287:203–9.
14. Jones, DP. Redox potential of GSH/GSSG couple: Assay and biological significance. Methods Enzymol. Academic Press Inc., 2002;348:93–112.
15. Kauffman, ME, Kauffman, MK, Traore, K, Zhu, H, Trush, MA, Jia, Z, et al. MitoSOX-Based Flow Cytometry for Detecting Mitochondrial ROS. React Oxyg species (Apex, NC). NIH Public Access, 2016;2:361.
16. Kundu, K, Knight, S, Willett, N, Lee, S, Taylor, W, and Murthy, N. Hydrocyanines: a class of fluorescent sensors that can image reactive oxygen species in cell culture, tissue, and in vivo. Angew Chem Int Ed Engl. Angew Chem Int Ed Engl, 2009;48:299–303.
17. Machado, MD, and Soares, E V. Assessment of cellular reduced glutathione content in Pseudokirchneriella subcapitata using monochlorobimane. J Appl Phycol. 2012;24:1509–16.
18. Mandavilli, B, and Janes, M. Detection of intracellular glutathione using ThiolTracker violet stain and fluorescence microscopy. Curr Protoc Cytom. Curr Protoc Cytom, 2010;Chapter 9:
19. McCord, JM, and Fridovich, I. Superoxide Dismutase. J Biol Chem. Elsevier, 1969;244:6049–55.
20. Miller, E, Tulyathan, O, Isacoff, E, and Chang, C. Molecular imaging of hydrogen peroxide produced for cell signaling. Nat Chem Biol. Nat Chem Biol, 2007;3:263–7.
21. Möller, MN, Rios, N, Trujillo, M, Radi, R, Denicola, A, and Alvarez, B. Detection and quantification of nitric oxide–derived oxidants in biological systems. J Biol Chem. 2019;294:14776–802.
22. Morrow, JD, Hill, KE, Burk, RF, Nammour, TM, Badr, KF, Roberts, LJ, et al. A series of prostaglandin F2-like compounds are produced in vivo in humans by a non-cyclooxygenase, free radical-catalyzed mechanism. Proc Natl Acad Sci U S A. National Academy of Sciences, 1990;87:9383.

23. Paital, B. A Modified Fluorimetric Method for Determination of Hydrogen Peroxide Using Homovanillic Acid Oxidation Principle. Biomed Res Int. Hindawi Limited, 2014;2014:.
24. Patton, GM, Fasulo, JM, and Robins, SJ. Analysis of lipids by high performance liquid chromatography: Part I. J Nutr Biochem. Elsevier, 1990;1:493–500.
25. Promega. GSH-Glo™ Glutathione Assay Instruc ons for Use of Products V6911 and V6912 [Internet].
26. Pryor, WA, Stanley, JP, and Blair, E. Autoxidation of polyunsaturated fatty acids: II. A suggested mechanism for the formation of TBA-reactive materials from prostaglandin-like endoperoxides. Lipids. Springer-Verlag, 1976;11:370–9.
27. Ren, W, and Ai, H-W. Genetically Encoded Fluorescent Redox Probes. Sensors (Basel). Multidisciplinary Digital Publishing Institute (MDPI), 2013;13:15422.
28. Roberts, LJ, and Morrow, JD. Measurement of F2-isoprostanes as an index of oxidative stress in vivo. Free Radic Biol Med. Pergamon, 2000;28:505–13.
29. Stankowski, JN, Codreanu, SG, Liebler, DC, and McLaughlin, B. Analysis of Protein Targets by Oxidative Stress Using the OxyBlot and Biotin–Avidin-Capture Methodology. Neuromethods. Humana Press, 2011;56:365–81.
30. Valavanidis, A, Vlachogianni, T, and Fiotakis, C. 8-hydroxy-2’ -deoxyguanosine (8-OHdG): A critical biomarker of oxidative stress and carcinogenesis. J Environ Sci Health C Environ Carcinog Ecotoxicol Rev. J Environ Sci Health C Environ Carcinog Ecotoxicol Rev, 2009;27:120–39.
31. Ward, M, Rego, A, Frenguelli, B, and Nicholls, D. Mitochondrial membrane potential and glutamate excitotoxicity in cultured cerebellar granule cells. J Neurosci. J Neurosci, 2000;20:7208–19.
32. Woolley, J, Stanicka, J, and Cotter, T. Recent advances in reactive oxygen species measurement in biological systems. Trends Biochem Sci. Trends Biochem Sci, 2013;38:556–65.
33. Yokoyama, C, Sueyoshi, Y, Ema, M, Mori, Y, Takaishi, K, and Hisatomi, H. Induction of oxidative stress by anticancer drugs in the presence and absence of cells. Oncol Lett. Spandidos Publications, 2017;14:6066–70.
34. Yuye Chen, Qingmei Zhong, Yilin Wang, Chunling Yuan, Xiu Qin, and Yuanjin Xu. Colorimetric detection of hydrogen peroxide and glucose by exploiting the peroxidase-like activity of papain. RSC Adv. Royal Society of Chemistry, 2019;9:16566–70.
35. Zhao, B, Summers, FA, and Mason, RP. Photooxidation of Amplex Red to resorufin: implications of exposing the Amplex Red assay to light. Free Radic Biol Med. NIH Public Access, 2012;53:1080.
36. Zielonka, J, Vasquez-Vivar, J, and Kalyanaraman, B. Detection of 2-hydroxyethidium in cellular systems: a unique marker product of superoxide and hydroethidine. Nat Protoc 2008 31. Nature Publishing Group, 2007;3:8–21.
37. Click-iT™ Lipid Peroxidation Imaging Kit - Alexa Fluor™ 488 [Internet]. Available from: https://www.thermofisher.com/order/catalog/product/C10446#/C10446.
