# Supplementary material for: Computational Models on Pathological Redox Signalling Driven by Pregnancy: A Review
Source: Antioxidants (Basel). 2022 Mar 18;11(3):585. doi: 10.3390/antiox11030585 (PMC8945226; doi:10.3390/antiox11030585)
Supplement: Supplementary file 1 [file antioxidants-11-00585-s001.zip › Supplementary material 2.pdf]

Table of Computational models of redox sensitive pathways

Reference	Model Description	New Insights	Limitations	Availability	Tool
**Atom**					
(16)	• Chemical/atom structures<br>• Quantum transport	Electron transport increases or is disrupted depending of zinc binding location	Difficult to integrate into bigger systems	Not open access	Transiesta 4.0 NWChem 6.4
(15)	• FAD in Cry1At<br>• Vertical energy gaps<br>• Vertical ionization energy<br>• Vertical electron affinity<br>• Gibbs free energy	• Accurate estimates of redox potentials of biological macromolecules<br>• Importance of long-range electrostatic interactions	Difficult to integrate into bigger systems	Equations and parameters available in supplementary material	BioEFP method Specifics unknown
**Kinetic, ODE, and Agent**					
(2)	• Modulation of ETC ROS production state 3 and 4 respiration<br>• Role of substrates and respiratory inhibitors<br>• Membrane potential<br>• ROS scavengers	• ROS levels are controlled by mitochondrial redox balance<br>• ROS production declines with decreasing membrane potential<br>• ROS increase when scavenging capacity is exhausted	• Conservative number of states<br>• Limited range of timescales<br>• Qi considered thermodynamically stable	http://icm.jhu.edu/models	MATLAB 7.1
(1)	• IL-4 network<br>• ODE including elementary mass action kinetics for all reactions<br>• pSTAT6 dynamics<br>• Systems model including ROS	• Reversible PTP oxidation is the primary redox regulatory mechanism in IL-4 redox pathway	• Specific to the IL-4 signaling pathway of the immune system<br>• Lack of compartmentation	https://simtk.org/projects/il4_redox	MATLAB Symbiology toolbox
(4)	• Rule based (ODE), CTMC<br>• ROS<br>• Lipid draft<br>• WNT signalling<br>• Considers spatial	• Characterisation of interplay between ROS and WNT signalling controlling nuclear beta-catenin levels	Neuron specific	Specifications in supplementary material	James II SESSL experiment
(9)	• Electron carriers for C1, C3, C4 + quantities<br>• Electron transfer network<br>• Reactions + rate equations<br>• pH<br>• Membrane potential	• Semiquinone of c1 may be ROS forming site<br>• Inhibition of c3 and 4 reduces ros production by c1 semiquinone<br>• Depolarization activates ros production by antimycin-inhibited complex3	Non stated	http://insysbio.ru	DBSolve Optimum software
(5)	• Agend based<br>• Intramitochondrial reactions<br>• Cytosolic activities	• NAD+ and ATP decline, and ROS decrease in an age dependent manner<br>• Effects of mitophagy	Based on c. *elegans*	Parameters and equations given in supplementa	AnyLogic multimethod simulation software GraphPad

	• Nuclear activities<br>• Mitochondrial stress-state phenotyping	• DAF16 and SKN1 expression required for healthy ageing		ry material	Prism 7.0
(8)	• Energetic-redox,<br>• Ionic processes<br>• pH regulation<br>• Transport between compartments	• SOD compartmentation and ROS generation can lead to mitochondrial chaotic dynamics<br>• SOD2 levels dictate chaotic, stable, and pathological function<br>• Hypothesized to lead to cardiac fibrillation	None stated	Available on request<br><br>Initial conditions available in supplementary material	MatCont 2.4 (MATLAB)<br><br>Time series of state variables - phase space reconstruction: MutualInfor 0.9 package<br><br>Lyapunov exponents: FET<br><br>Power Spectrum Analysis Fast Fourier Transform
(10)	• Core motif<br>• Active Prx 3<br>• Inactive Prx3<br>• Mitochondrial H_2O_2<br>• Cytosolic H_2O_2<br>• Mitochondrial Srx<br>• Reaction rates	delay in mitochondrial Srx import, in combination with separation of fast and slow reactions, is sufficient to generate self-sustained relaxation-like oscillations	Parameters differs between cell and tissue types	Available in supplementary material	Python - odeint (scipy)
(11)	• Kinetic /network<br>• Peroxiredoxins<br>• Thioredoxins	*in vivo* thioredoxin oxidation measures can be used as surrogate indicators for flux	Allows qualitative (not quantitative) comparisons	Equations and parameters stated directly in the article	PySCeS Copasi
**Network based**					
(17)	• ROS-induced ROS release based on reaction-diffusion<br>• Network of individual mitochondria including ROS dependent mechanisms<br>• Membrane potential	Clusters of oscillating mitochondria can lead to cell death and progression of heart disease	• Cardiomyocyte specific<br>• Does not reproduce entirety of network properties<br>• 2D, however O_2 can diffuse in three dimensions	Parameters and equations given in supplementary material	CVODE (ODE solver)
(13)	• Boolean network model<br>• Oxidative stress response<br>• PI3k/Akt pathway apoptosis<br>• Nrf2-Keap1 signalling	New approach to pinpoint fault locations by using temporal variations in oxidative stress input	None stated	Not open access	MATLAB

(12)	• Transcriptional feedback through FOXO<br>• Interaction with ROS<br>• Insulin signalling<br>• ODE	• Long term dynamics of insulin<br>• Describes the concentration dependent regulatory effect of oxidative stress on insulin signalling	• None stated<br>• Author suggested extension of model including glycolysis, oxidative phosphorylation, and ROS production	Biomodels MODEL1212210000	SBML, Copasi v4.6 R v2.9.2
(6)	• SBML<br>• Oxidative stress<br>• TGFbeta<br>• IL1<br>•	Highlighted the role of oxidative stress in cartilage breakdown associated with ageing	Specific to ageing	Biomodels MODEL1402200004	CellDesigner COPASI
(3)	• Network based<br>• FDX, FTR, FNR<br>• NADPH, NTRC pathway<br>• Based on previously published models	• Describes functional state of chloroplast thiol regulatory network<br>• The stromal redox state is H_2O_2 dependent	Specific to chloroplast metabolism	provided in supplementary material	MATLAB
(7)	• Kinetic metabolism model CardioGlyco<br>• Redox cofactors<br>• Metabolites and equations of interactions	Phosphoglucose isomerase activity affects myocyte growth via mTOR activity	Cardiomyocite specific	Biomodels: MODEL1910170001	COPASI
**ML**					
(14)	• Sequential distance<br>• Position-specific scoring matrix profile<br>• Predicted secondary structure<br>• Predicted solvent accessibility<br>• Physical chemical property<br>• ML: SVM	• Prediction of redox-sensitive cysteines<br>• Independent of protein structure data	None stated	http://biocomputer.bio.cuhk.edu.hk/RSCP Does not seem to be supported	PSI-Blast SSpro Matlab: Libsvm 3.2 + svm-rfe R - nnet

Table of computational models of redox sensitive pathways including reference, model components (description), major new insight, limitations as stated by the author(s), availability, and tools used to create and apply each model. Stated availability “in supplementary material” refers to the original publication of the model, stated in references (ML: machine learning).

Bibliography

1. Dwivedi, G, Gran, MA, Bagchi, P, and Kemp, ML. Dynamic Redox Regulation of IL-4 Signaling. PLoS Comput Biol. Public Library of Science, 2015;11:.
2. Gauthier, LD, Greenstein, JL, Cortassa, S, O'Rourke, B, and Winslow, RL. A Computational model of reactive oxygen species and redox balance in cardiac mitochondria. Biophys J. The Biophysical Society, 2013;105:1045–56.
3. Gerken, M, Kakorin, S, Chibani, K, and Dietz, K-J. Computational simulation of the reactive oxygen species and redox network in the regulation of chloroplast metabolism. PLOS Comput Biol. Public Library of Science, 2020;16:e1007102.
4. Haack, F, Lemcke, H, Ewald, R, Rharass, T, and Uhrmacher, AM. Spatio-temporal Model of Endogenous ROS and Raft-Dependent WNT/Beta-Catenin Signaling Driving Cell Fate Commitment in Human Neural Progenitor Cells. PLOS Comput Biol. Public Library of Science, 2015;11:e1004106.
5. Hoffman, TE, Barnett, KJ, Wallis, L, and Hanneman, WH. A multimethod computational simulation approach for investigating mitochondrial dynamics and dysfunction in degenerative aging. Aging Cell. Blackwell Publishing Ltd, 2017;16:1244–55.
6. Hui, W, Young, DA, Rowan, AD, Xu, X, Cawston, TE, and Proctor, CJ. Oxidative changes and signalling pathways are pivotal in initiating age-related changes in articular cartilage. Ann Rheum Dis. BMJ Publishing Group Ltd, 2016;75:449–58.
7. Karlstaedt, A, Khanna, R, Thangam, M, and Taegtmeyer, H. Glucose 6-Phosphate Accumulates via Phosphoglucose Isomerase Inhibition in Heart Muscle. Circ Res. Lippincott Williams and Wilkins, 2020;126:60–74.
8. Kembro, JM, Cortassa, S, Lloyd, D, Sollott, SJ, and Aon, MA. Mitochondrial chaotic dynamics: Redox-energetic behavior at the edge of stability. Sci Reports 2018 81. Nature Publishing Group, 2018;8:1–11.
9. Markevich, NI, and Hoek, JB. Computational modeling analysis of mitochondrial superoxide production under varying substrate conditions and upon inhibition of different segments of the electron transport chain. Biochim Biophys Acta - Bioenerg. Elsevier, 2015;1847:656–79.
10. del Olmo, M, Kramer, A, and Herzel, H. A robust model for circadian redox oscillations. Int J Mol Sci. MDPI AG, 2019;20:.
11. Padayachee, L, Rohwer, JM, and Pillay, CS. The thioredoxin redox potential and redox charge are surrogate measures for flux in the thioredoxin system. Arch Biochem Biophys. Academic Press, 2020;680:108231.
12. Smith, GR, and Shanley, DP. Computational modelling of the regulation of Insulin signalling by oxidative stress. BMC Syst Biol. BioMed Central, 2013;7:41.
13. Sridharan, S, Layek, R, Datta, A, and Venkatraj, J. Boolean modeling and fault diagnosis in oxidative stress response. BMC Genomics. BMC Genomics, 2012;13 Suppl 6:
14. Sun, M, Zhang, Q, Wang, Y, Ge, W, and Guo, D. Prediction of redox-sensitive cysteines using sequential distance and other sequence-based features. BMC Bioinforma 2016 171. BioMed Central, 2016;17:1–10.
15. Tazhigulov, RN, Gurunathan, PK, Kim, Y, Slipchenko, L V., and Bravaya, KB. Polarizable embedding for simulating redox potentials of biomolecules. Phys Chem Chem Phys. Royal Society of Chemistry, 2019;21:11642–50.
16. Yu, J, Horsley, JR, and Abell, AD. Unravelling electron transfer in peptide-cation complexes: a model for mimicking redox centres in proteins. Phys Chem Chem Phys. Royal Society of Chemistry, 2020;22:8409–17.
17. Zhou, L, Aon, MA, Almas, T, Cortassa, S, Winslow, RL, and O'Rourke, B. A Reaction-Diffusion Model of ROS-Induced ROS Release in a Mitochondrial Network. PLOS Comput Biol. Public Library of Science, 2010;6:e1000657.
