# Supplementary material for: Computational Models on Pathological Redox Signalling Driven by Pregnancy: A Review
Source: Antioxidants (Basel). 2022 Mar 18;11(3):585. doi: 10.3390/antiox11030585 (PMC8945226; doi:10.3390/antiox11030585)
Supplement: Supplementary file 1 [file antioxidants-11-00585-s001.zip › Supplementary material 4 (2).pdf]

Use Case of Redox Map on Minerva

Example session using public data set GSE177049 containing both miRNA and gene expression data. Starting with gene expression; 3113 probes were mapped, most of which lie in the molecular interaction map (MIM) and not on the visible map. Nevertheless, some genes and their regulation (up = red, down = blue) are **visualized directly,** giving the user an intuitive overview of how their data may be interpreted (Figure 1).

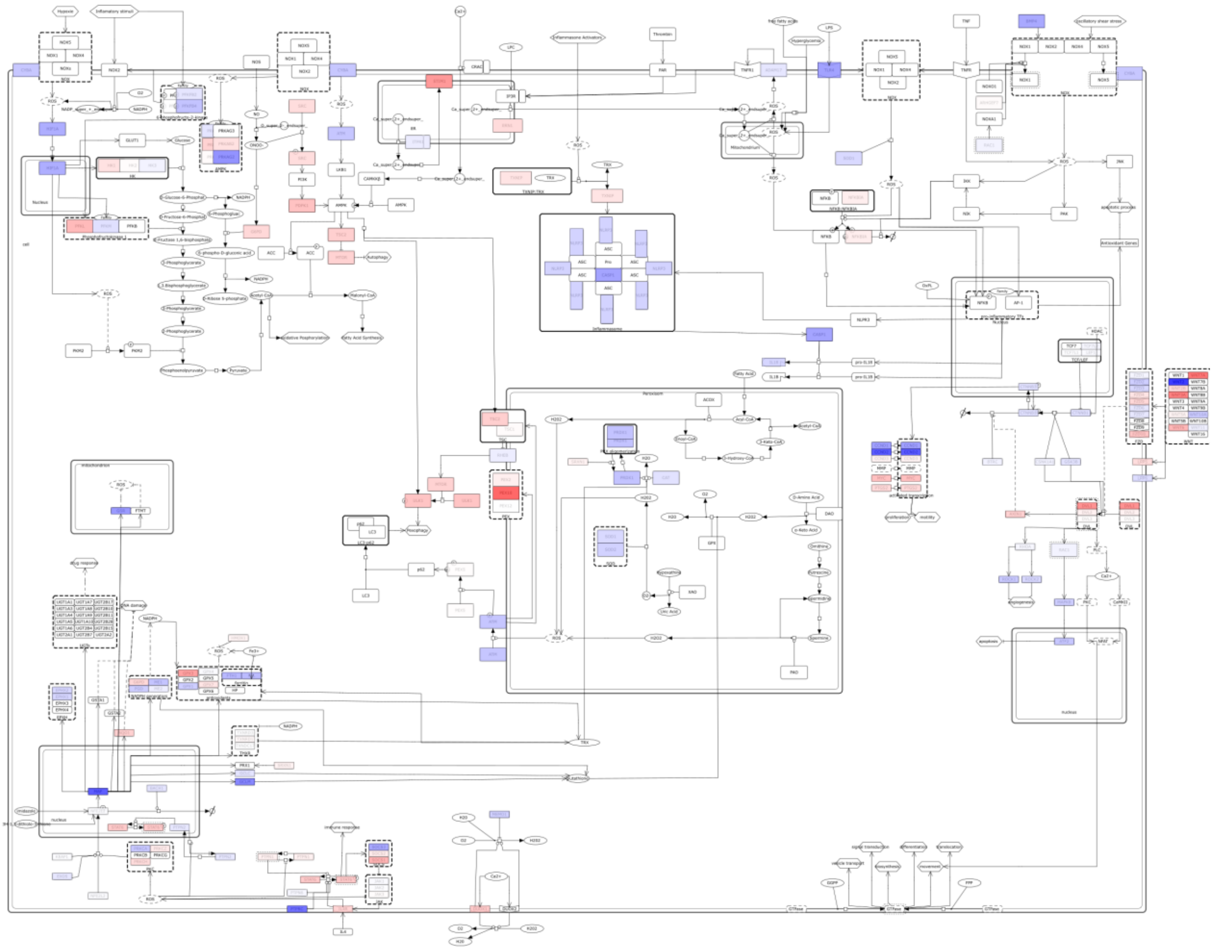

Figure 1: Gene expression overlay on redox map uploaded on Minerva. Compartments are spatial compartments. Their borders can be considered membranes and are visualized as such. Rectangles are proteins while thick black bordered rectangles are complexes. Arrows type indicate inhibition, modulation, activation, transport, and state transition. The overlay indicates protein up (red) or down (blue) regulation. A complete legend is available under https://air.bio.informatik.uni-rostock.de/

This redox map does not contain miRNA on the visual layer, hence direct visualization of miRNA expression data is not possible**.** However, phenotype- including predicted level of phenotype change and significance prediction caused by deregulated miRNA is possible and visualized on the map in a similar manner (Figure 2).

A

Phenotype	Level (-1 to 1)	P value
autophagy	-0.46	0.2879
oxidative posphorylation	0.51	0.1225
pexophagy	-0.23	0.5018
fatty acid synthesis	0.5	0.1270
apoptotic process	-0.57	0.1402
cell movement	-0.53	0.1251
drug response	0.84	0.0208*
dna damage	-0.84	0.0210*
immune response	0.21	0.6122
proliferation	1	0.0049**
cell motility	1	0.0049**
angiogenesis	-1	0.0092**
apoptosis	-0.98	0.0138*

B

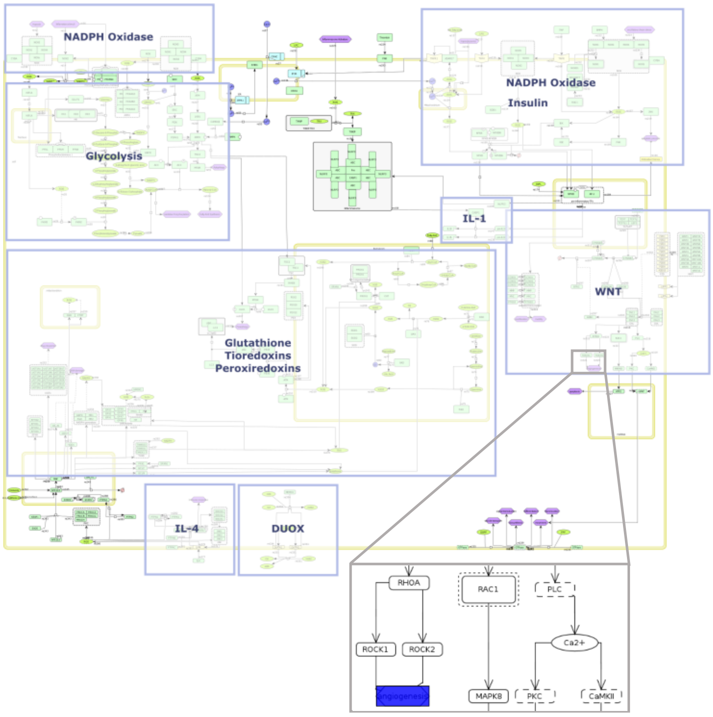

Figure 2: Phenotype prediction based on miRNA expression data (A) and example visualization of resulting phenotype within the redox map (B). Phenotype prediction includes predicted levels of phenotype ranging from -1 to 1 and p value. On the map the predicted phenotype level is color coded (blue = downregulated).

This type of analysis, based on experimental data and then validated using independent follow up experiments can lead to new biomarker identifications.
